# Surface rupture and landscape response in the middle of the great Mw 8.3 1934 earthquake mesoseismal area: Khutti Khola site

**DOI:** 10.1038/s41598-023-30697-7

**Published:** 2023-03-20

**Authors:** Magali Riesner, Laurent Bollinger, Magali Rizza, Yann Klinger, Çağıl Karakaş, Soma Nath Sapkota, Chanda Shah, Cyrielle Guérin, Paul Tapponnier

**Affiliations:** 1 CEA, DAM, DIF, 91297 Arpajon, France; 2 Present Address: Aix Marseille Univ, CNRS, IRD, Coll. France, CEREGE, Aix‐en‐Provence, France; 3 Aix Marseille Univ, CNRS, IRD, Coll France, CEREGE, Aix‐en‐Provence, France; 4 Institut de Physique du Globe de Paris, CNRS, Université Paris Cité, Paris, France; 5 Schlumberger Stavanger Research Center, Risabergvegen 3, 4056 Tananger, Norway; 6 Department of Mines and Geology, Kathmandu, Nepal; 7 Institute of Crustal Dynamics, China Earthquake Administration, Beijing, China

**Keywords:** Natural hazards, Tectonics

## Abstract

Large earthquakes breaking the frontal faults of the Himalayan thrust system produce surface ruptures, quickly altered due to the monsoon conditions. Therefore, the location and existence of the Mw8.3 1934 Bihar–Nepal surface ruptures remain vividly disputed. Even though, previous studies revealed remnants of this surface rupture at the western end of the devastated zone, ruptures extent remains undocumented in its central part. Evidence for recent earthquakes is revealed along the frontal thrust in this region. The Khutti Khola river cuts an 8 m-high fault scarp exposing Siwalik siltstone thrusted over recent alluvial deposits, with faults sealed by a colluvial wedge and undeformed alluvial sediments. Detrital charcoals radiocarbon dating reveals that the last event occurred between the seventeenth century and the post-bomb era, advocating for the 1934 earthquake as the most recent event. In the hanging wall, fluvial terraces associated with fault scarps were abandoned after a penultimate event that happened after the tenth century, a rupture we associate with the historic earthquake of 1255CE. Slips of 11–17 m and 14–22 m for the 1934 and 1255 earthquakes, respectively, compare well with the ~ 10–15 m slip deficit accumulated between the two earthquakes, suggesting that most of the deformation along the front is accommodated by surface-rupturing earthquakes.

## Introduction

Large earthquakes along thrust systems transfer towards the surface large amounts of seismic slip accumulated at mid-crustal depth during centuries^1–7^. The thrusting of the hanging wall by several meters during those large events is often accompanied by dramatic surface breaks^8–10^ that can be distributed along several parallel strands (e.g.^11^). Locally, the expression of surface ruptures can be highly variable, implying frequent hanging wall collapses, fault-related fold scarps, or pressure ridges^12–16^, which complicates the quantification of displacement. In the case of historical and paleo-earthquakes ruptures along megathrusts systems, identifying and quantifying surface ruptures are even more difficult. Local climatic conditions can lead to rapid erosion, or even complete removal, of the primary surface ruptures. After a large earthquake, the fault trace documentation is hampered by several processes such as widespread landslides covering the surface rupture (e.g.^17,18^). In addition, off-fault landslides in the hanging wall can deliver large amount of excess sediment to rivers^19^, which are transported downwards to the footwall and contribute to significant aggradation along the thrust system front, locally burying the fault scarps^20^.

**Figure 1:**
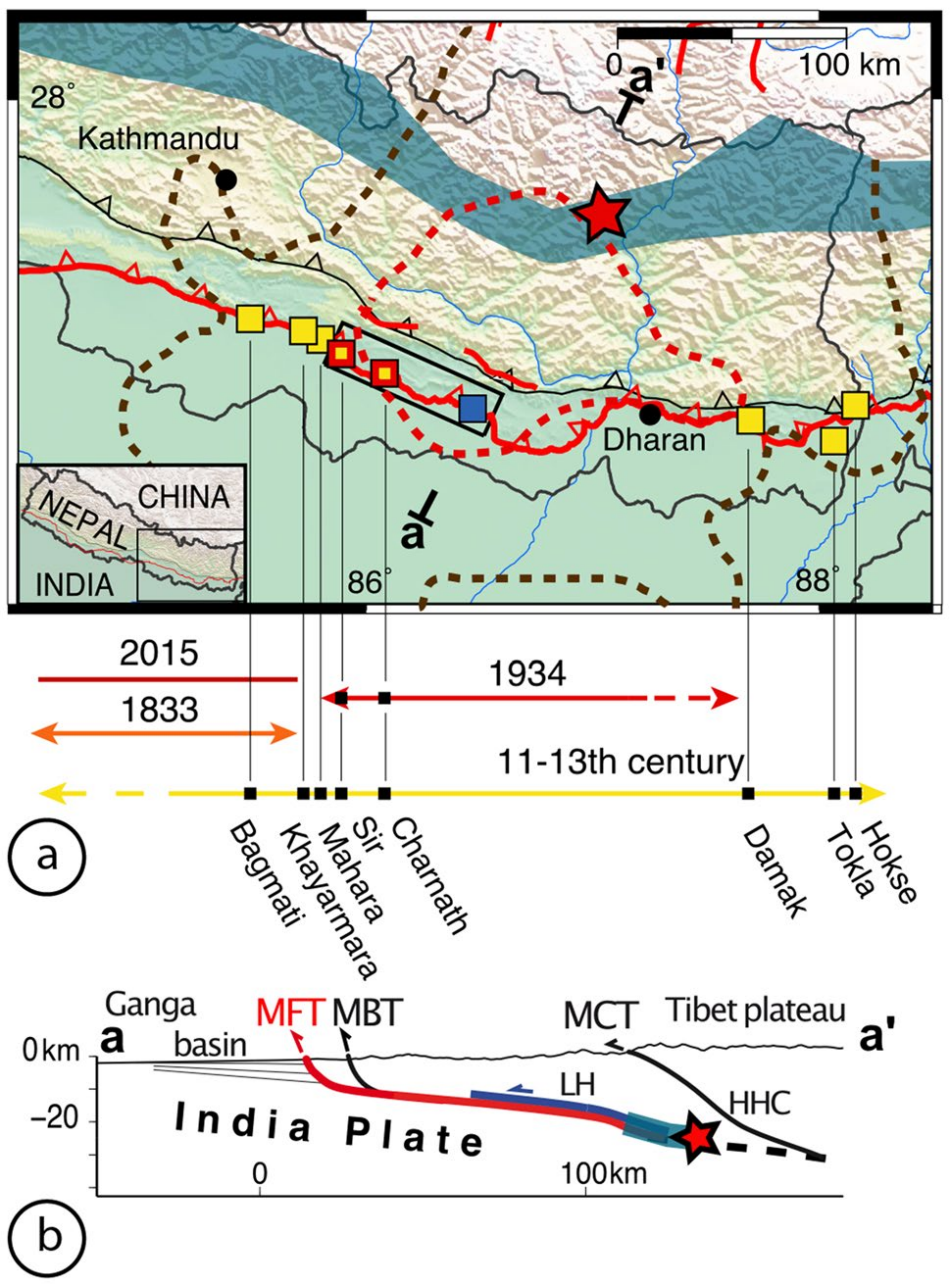
Seismotectonic map of central-eastern Nepal. (**a**) Yellow and red squares locate the paleoseismic sites excavated through paleoruptures dating from medieval times and the surface rupture of the 1934 earthquake, respectively^20–22,24,26–28,34^). Blue square: Khutti Khola trench site. Red star locates the instrumental epicenter of the 1934 Bihar–Nepal earthquake^35^. Brown and red dashed lines represent respectively the isoseists of intensities VII and VIII corrected for liquefaction effects^32,33^. Black rectangle locates Fig. 2. (**b**) Cross section a–a′ across the range in eastern Nepal. Partial and total ruptures of the Main Himalayan Thrust are schematically represented in blue and red respectively. *MFT* Main Frontal Thrust, *MBT* Main Boundary Thrust, *MCT* Main Central Thrust, *MHT* Main Himalayan Thrust, *LH* Lesser Himalaya, *HHC* High Himalaya Crystalline. Figure generated with ENVI Classic (https://www.l3harrisgeospatial.com/Software-Technology/ENVI), Adobe illustrator CC (http://www.adobe.com/fr/products/illustrator.html) and MAPublisher 10.8.1.789.

In the Himalaya, the strain accumulated along the Main Himalayan Thrust fault (MHT) can be either partially or completely released by megathrust earthquakes that rupture only the lower part of the MHT, or the entire MHT up to the surface at the Main Frontal Thrust (MFT) (Fig. 1). It is generally accepted that megathrust earthquakes on the MHT (Mw > 8) have ruptured the surface along the MFT^9,20–31^. In the case of the 1934 earthquake, however, massive destruction and numerous landslides were described in the historical record, but no surface rupture was identified at the time^18^. Despite this lack of early observations, recent paleoseismic studies at Sir Khola^23^ and Charnath Khola^20^ (~ 25 km apart) argue for a surface rupture associated with this earthquake at the western edge of the mesoseismal area (intensity VII–VIII) documented by the macroseismic intensities (Fig. 1^32,33^).

However, persistent ambiguity about the mere existence of a surface rupture, and its possible length, for the 1934 earthquake has fueled opposite interpretations concerning (1) the location of the Bihar–Nepal earthquake rupture and (2) the possibility that megathrust earthquakes (Mw > 8) might be mainly blind^21^, as illustrated by ongoing argument about interpretation of outcrop at the Sir Khola river site^23,36^.

**Figure 2:**
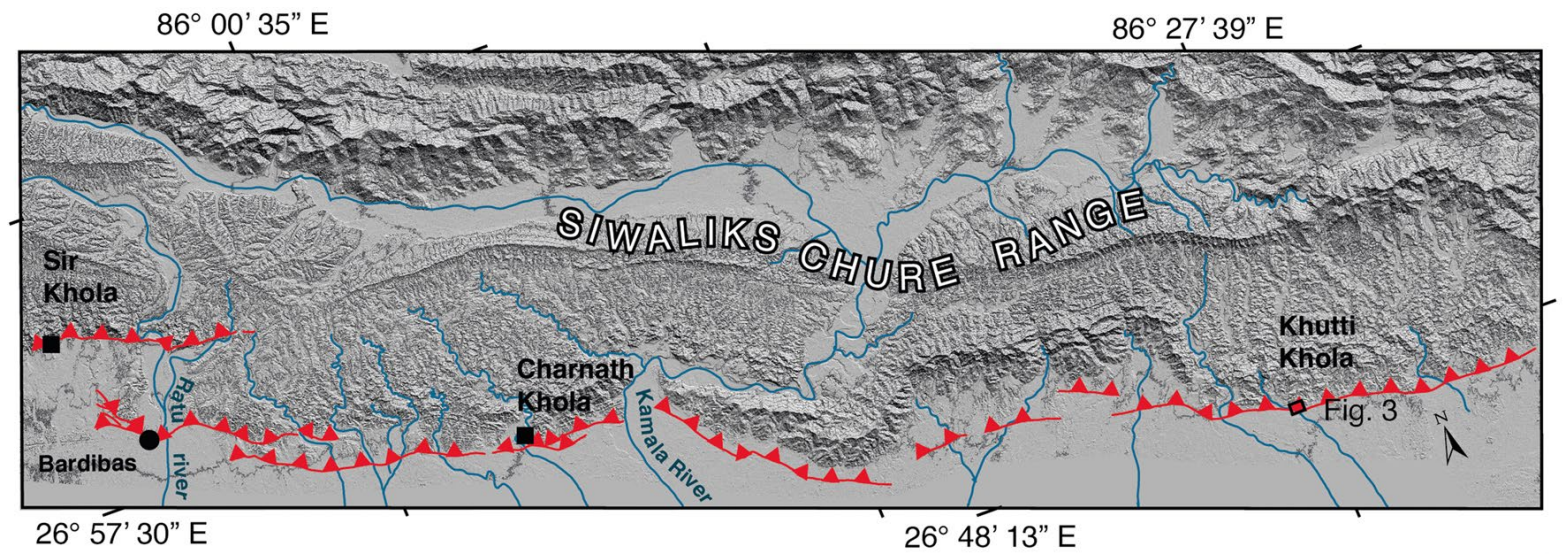
Map of the Main Frontal Thrust strands (red), between the Sir and the Khutti Khola rivers based on ALOS 5 m resolution digital elevation model. Small black boxes locate the sites studied in the area. ALOS 5 m DEM. Figure generated with ENVI Classic (https://www.l3harrisgeospatial.com/Software-Technology/ENVI), Adobe illustrator CC (http://www.adobe.com/fr/products/illustrator.html) and MAPublisher 10.8.1.789.

Here we focus our paleoseismic study on a site located in the middle of the mesoseismal area (intensity VIII), in order to re-asses the existence and characteristics of the Bihar–Nepal earthquake surface rupture and to document potential earlier earthquakes. New data from morphological study and paleoseismic trenching along the Khutti Khola river (Fig. 2) are presented below (see the “Methods” section at the end). The implications of these findings are then discussed to provide a chronological tectonic evolution at this site and a better understanding of scarp preservation in the Himalaya.

## Morphotectonic settings at the Khutti Khola river

**Figure 3:**
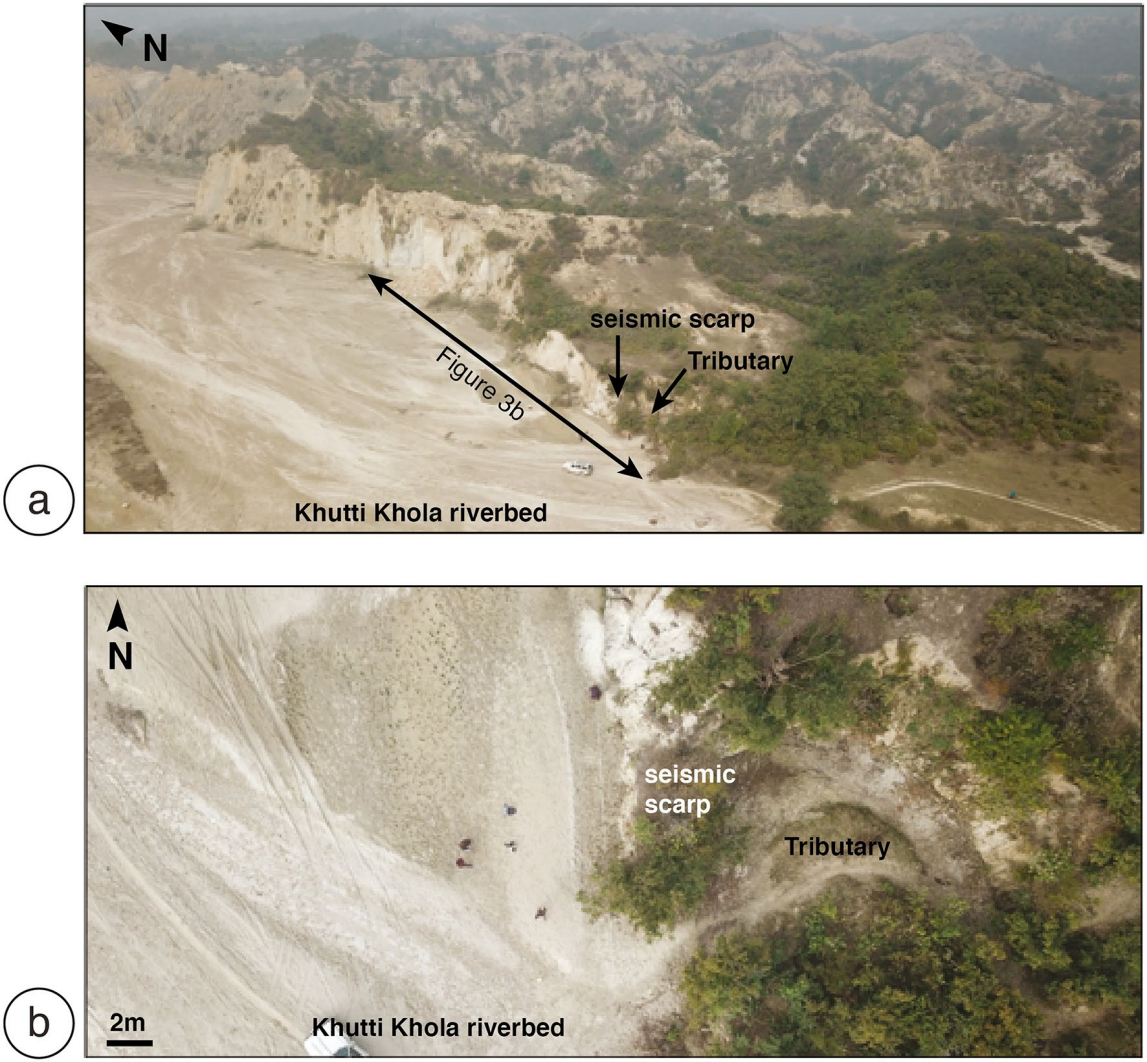
(**a**) Perspective view of the Khutti khola site locating the rivercut section Fig. 4b, (**b**) map view.

**Figure 4:**
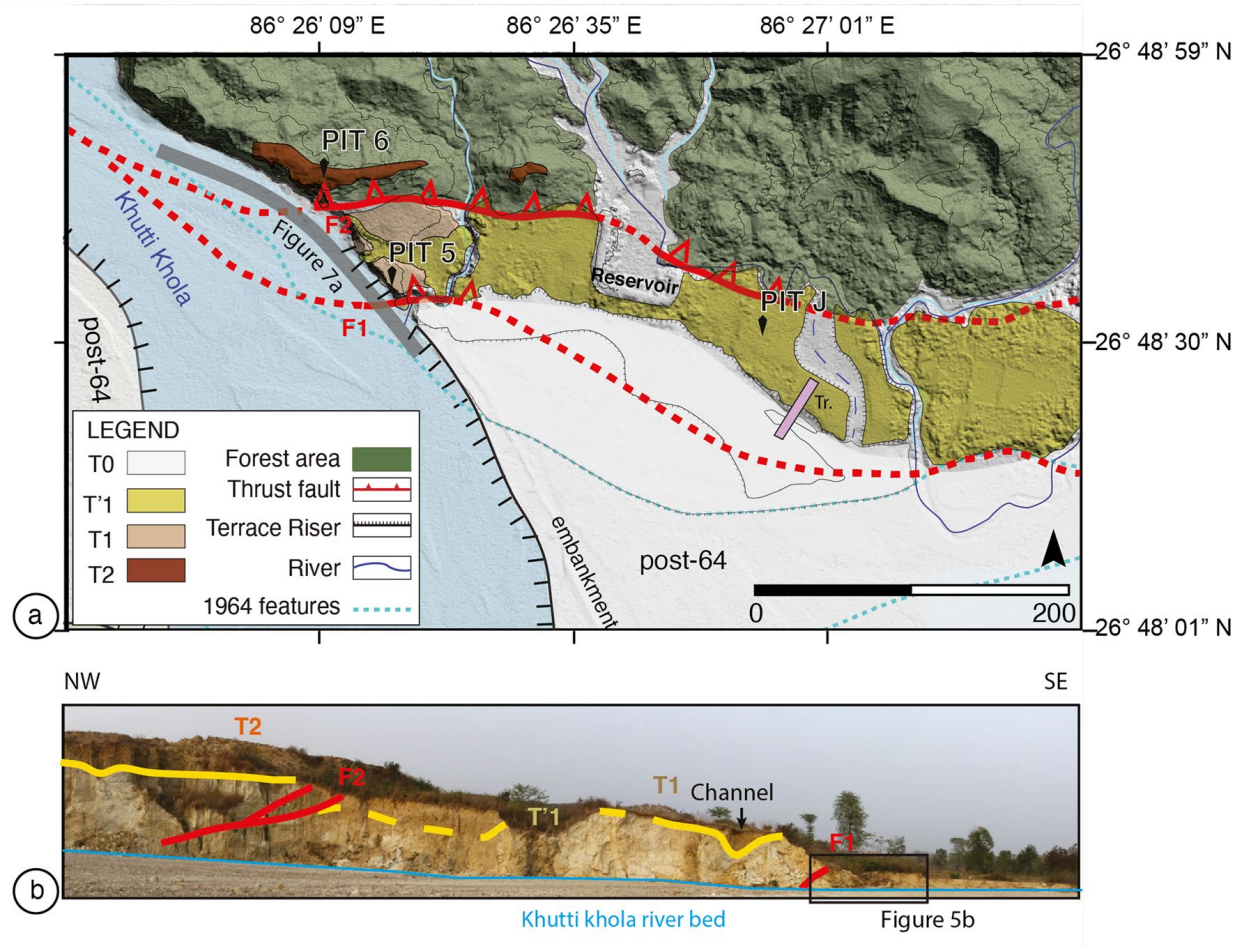
(**a**) Map of the active tectonics and geomorphology of the Khutti Khola site with the Main Frontal Thrust in red (see location Fig. 2). Grey line locates the river cut Fig. 7a. *Tr.* Trench with undeformed units. (**b**) Rivercut picture with the main geological features, the faults and the vertical offset between the current riverbed and the top of terraces T1 and T2. Yellow line represent the top of the Siwalik. Sampled pit and channel are logged in Fig. S3 from the Supplementary Materials, the black box locates the trench site. Figure generated with ENVI Classic (https://www.l3harrisgeospatial.com/Software-Technology/ENVI), Adobe illustrator CC (http://www.adobe.com/fr/products/illustrator.html) and MAPublisher 10.8.1.789.

The main topographic front of the Siwalik hills in the Khutti Khola area is relatively linear, following a major fault, with a simpler geometry than along the Sir-Bardibas section (Figs. 2, 3, 4a). The regional strike of the frontal fault is N110. It has been imaged at depth by several seismic reflection profiles that evidence a ~ 30°–45° north dipping frontal thrust in the area^37,38^.

The Khutti Khola river catchment at the topographical front (86.454° E/26.796° N) is about 40 km^2^ (Fig. 2). Remnants of frontal thrusts with well-preserved evidence of recent faulting and abandoned fluvial terraces are still exposed in one rivercut along the east bank of the river.

This ~ 150 m-long ~ 30 m-high natural rivercut cliff exposes predominantly light-colored siltstones stacked on top of each other and separated by two north-dipping thrust faults (F1 and F2 Fig. 4). Both thrusts bring sheared Siwalik siltstones on top of alluvial deposits. To the north, the ~ 20° N-dipping F2 fault reaches the surface at the toe of a 7 m-high scarp bordering the ~ 4°–5° S-dipping T2 tread. This fault places Siwalik siltstones on top of the alluvial deposits composing T1. T2 is composed of a ~ 5 m-thick sand-based alluvial deposits. The most frontal ~ 42° N-dipping F1 fault emerges at the toe of a ~ 8 m-high escarpment, below the gently S-dipping T1 tread (~ 3°). Terrace T1 is made of fine fluvial material with a variable thickness, reaching 10 m on its northern edge, although considerably thinner closer to F1. Thirty meters north of F1, the western terrace raiser of T1 exposes a lateral channel mapped as surface T′1 farther east (in yellow in Fig. 4a).

A 2 m-large tributary flows intermittently during the summer monsoon at the toe of the 8 m-high frontal scarp (Figs. 3b, 4), where undeformed sediments are resting on the top of the T1 material. To the east of the river cut, the lateral extension of F1 has been eroded by a paleochannel of the Khutti Khola river, which was latter backfilled during some aggradation episode (Fig. 4).

In order to obtain stratigraphic and chronological constraints on the morphotectonic events at this site, we refreshed the natural rivercut in the vicinity of the F1 fault-plane and we excavated a trench, and several pits within the abandoned fluvial terraces. Detrital charcoals were sampled for radiocarbon dating.

## Paleoseismic observations in the Khutti Khola trench and chronological constraints on the trench sedimentary units

### Description of the stratigraphic units and thrust faults

**Figure 5:**
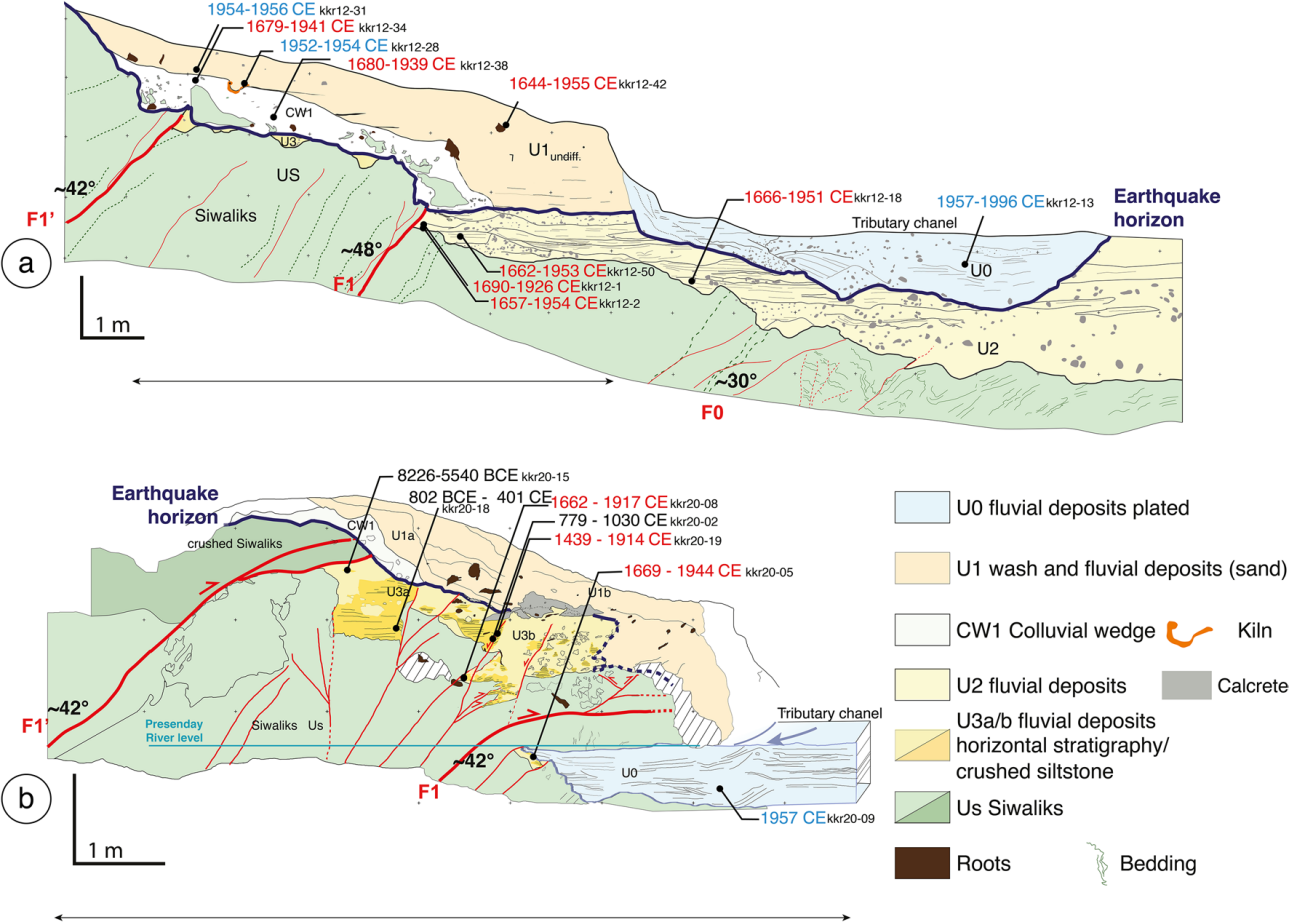
Simplified structural and stratigraphic logs of the rivercut and trench excavated in 2012 (**a**-Top) and refreshed in 2020 (**b**-bottom) with faults and individually calibrated detrital charcoal ages. See text for description of the stratigraphic units and discussions. See also the photomosaics in the supporting information data and detailed photos Figs. S1, S2, S3 and S4. Ages in blue when modern (postbomb), in black when interpreted as inherited, in red elsewhere.

In 2012 we refreshed, and logged the southern section of the natural rivercut cliff and excavated a trench at its toe (Fig. 5a, Fig. S1 in the Supplementary Data material). The western wall of the trench, opposite to the rivercut was not mapped, as it only exposed the lowermost base units. Refreshing and logging of the rivercut were complemented in 2020 (Fig. 5b, Figs. S2, S3, S4 in the Supplementary Data materials). The differences of outcrop exposure were significant after only 8 years (see Fig. 3). The first trench, excavated in 2012, was 19 m-long, 3 m-wide and 6.5 m-high. The excavation in 2020 was shallower, re-exhuming mainly the most superficial units affected by the deformation. Figure 5a,b report the logs of the 2012 and 2020 trenches’ wall, respectively. Photomosaics of the trenches and detailed photos can be found in the Supplementary Data materials (Figs. S1, S2, S3, S4).

In both trenches, the rivercut exposes bedrock composed of crushed Siwalik siltstones (Us) thrusted over alluvial sediments by several splay faults, with the two major faults F1 and F1′ dipping northward at ~ 45°. On the southern side of the trench, the uppermost unit of the wall (U0) is observed in both trenches in the prolongation of the tributary channel from the east. Unit U0 is composed of a ~ 1 m-thick alternation of brown gravels and yellow coarse sand layers and mostly horizontal interbedded paleo-channels. In 2012 we observed that unit U0 was deposited on top of a ~ 2.5 m-thick alluvial unit (U2) composed of laminated coarser gravel and pebble layers (20 to 5 cm-thick) with coarse sandy layer (20 to 30 cm-thick) and with heterogeneous material at its base. U2 presents an erosive base contact and is deposited on top of highly deformed, sheared and faulted Siwalik (F0, Fig. 5a). On the 2020 wall, U0 is plastered in front of U3 (a,b) and is also deposited directly on the deformed Siwalik as observed on the lower part of the wall (Fig. 5b).

In the northern side, the 2012 and 2020 logs show a rather different organization of deposits. On both logs, the topmost unit, U1 is undeformed and composed of fine light-brown sandy fluvial deposits (50 to 100 cm-thick). In 2020’s log, U1 is subdivided into two sub-units with at the base a light beige indurated fine sandy 10 to 30 cm-thick fluvial deposits (U1a). U1a is deposited on top of a thin (20–40 cm) colluvial wedge CW1 constituted of disorganized angular Siwalik pebble and cobble-size clasts within a brownish coarse sand indurated matrix. In the 2012’s log, U1 is similarly deposited on top of a well-preserved (1 m-thick, 6 m-long) colluvial wedge and wash (also named CW1). On this log, CW1 is composed of pebble- to boulder-sized angular Siwalik blocks embedded within a fine light-brown sandy matrix. On both 2012 and 2020 logs, the collapse wedge units are deposited on top of a fluvial unit U3. However, in the 2012 wall, U3 is poorly preserved and only found at the base of CW1, in small pockets within the eroded paleotopography of the Siwalik bedrock (Us). This U3 unit is wider (50–70 cm-thick) on the 2020 wall and is composed of two deformed and faulted sub-units logged as crushed indurated light-brown siltstone (U3a) and sandy fluvial deposits with a clear horizontal bedding (U3b). On both logs, U3 is deposited on top of the crushed and faulted Siwalik (Us) to the northern and southern sides of the walls, respectively. At the base of the trenches, Us is successively composed from north to south by (1) highly crushed, (2) faulted and massive, and (3) highly deformed and faulted Siwalik, overthrusting U2 and U3. The main faults affect Us, U3 and U2 and dip between ~ 30° and ~ 48° north. Several minor structures accommodate centrimetrical offsets (Fig. 5b). On the 2020 log, the bottom of U3 is uplifted by ~ 1.5 m in total by several faults.

### Radiocarbon dating of detrital charcoal samples from the stratigraphic sequence and the colluvial wedge

**Figure 6:**
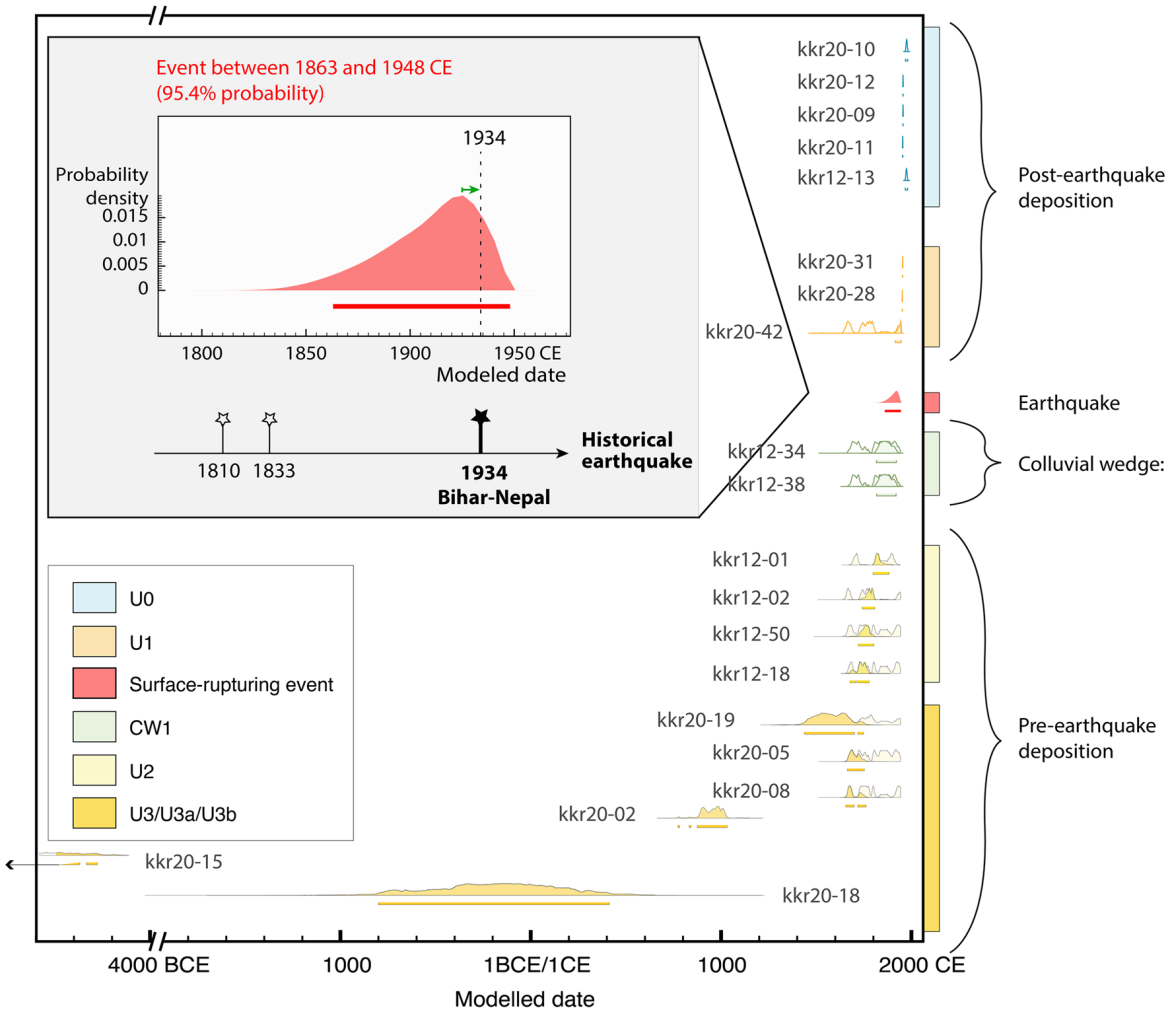
Oxcal chronostratigraphic sequence of the detrital charcoals built from their stratigraphic information. In this model we consider that reworking, inbuilt ages for charcoal samples (i.e., age of the wood at the time of burning) and transport time before deposition are negligible. Detrital charcoal radiocarbon ages are calculated using Oxcal 4.2.4^39^ chronostratigraphic models with IntCal13 atmospheric calibration curve^40^. Light and dark grey probability spectra, respectively, indicate the individually calibrated and modelled ages. Red probability spectra indicate the modelled ages for earthquake E1 estimated to fall between 1863 and 1948.

Twenty detrital charcoals were dated from 54 samples collected in 2012 and 2020 (Figs. 5, 6, Figs. S1, S2 of the Supplementary Data materials). The radiocarbon dates range from 8226BCE to 1996CE. The detrital charcoals radiocarbon ages sampled within U3 and U2 attest for their deposition after the mid seventeenth century. Indeed, half of the charcoals found in U3 could be as recent as the early twentieth century, while two of them—KKR20-05 and 08—were deposited after 1662CE. The three oldest charcoals (KKR20-02, -15 and -18) from that unit give older inconsistent dates and were discarded due to inheritance. All the ages obtained for the charcoals found at the base of U2 (i.e. KKR12-02, -18, -50) indicate that this unit was necessarily deposited after the mid seventeenth century.

Both units U2 and U3 have been affected by the overthrusting of the Siwalik slivers (Us) and more generally by penetrative deformations associated to the activation of the frontal thrust fault system during the last earthquake that occurred after the mid seventeenth century. This conclusion is supported by the ages obtained in colluvial wedge CW1. In fact, the calibrated ^14^C ages of the 2 charcoals found in the colluvial wedge (KKR12-34, -38) are, at a first-order, in the same age ranges than charcoals found in U3 and U2, implying that CW1 was deposited after 1680CE.

The three ages obtained for the charcoals sampled above the colluvial wedge in Unit U1, within a kiln or in the associated charcoal horizon at its base (KKR12-28, -31, -42) indicate that this unit was deposited after 1954CE. This unit was then incised by the tributary channel U0, which was active after 1957 CE (KKR12-13; KKR20-09), and before 1964CE when the tributary channel was documented on the first aerial photographs.

This chronology was refined by including stratigraphic information into the ^14^C age calibration process, using the Bayesian Oxcal calibration software^41^. Several models for the two trenches taken independently or combined were tested*.* Units U0, U1 and U2 are set as organized sequences. CW1 and U3 are set as unordered phases. The model presented on Fig. 6 takes into account a trench sequence as follows: U3, U2, CW1, U1, U0, assuming that the detrital charcoals sampled within the Colluvial Wedge CW1 predate the earthquake (as detailed in Ref.^20^). As noted above, the three oldest charcoals from U3 are considered to be inherited (8226-5540BCE, 802BCE-401CE, 779-1030CE for KKR20-15, -18 and -02, respectively), as they yield disparate ages significantly older than the fifteenth to twentieth century ages found elsewhere in U3 and U2. The 95.4% PDF (probability density function) for the age of the event associated with the collapse of the wedge CW1 in our model combining the 2012 and 2020 samples ranges from 1863 to 1948CE with the highest probability around 1923CE. This model does not account for any charcoal in-built age nor transport time lag (see Fig. 6^20,42^ and “Methods” section for more explanation).

## Discussion

### Age of the paleoearthquakes and terrace abandonments

Our study shows that the frontal thrust fault in the Khutti Khola outlet was activated after the mid seventeenth century, most probably between the mid nineteenth and mid twentieth century (Fig. 6).

During this period, 5 destructive earthquakes were documented in Kathmandu by chroniclers, in 1680, 1808, 1833, 1866 and 1934^42,43^. Of these earthquakes, only the 1934 earthquake caused damage associated with a macroseismic intensity greater than VII in the Siraha district, in which our site is located. In 1934, the area, in the core of the mesoseismal region, was devastated: Muksar and Udaipur Garhi, villages located respectively 6 km West and 17 km North of our site were affected by intensities VIII^33^.

Although similar high intensities were also reached later in the area, during the Udaypur August 20th 1988 earthquake^44^, the deep source of this earthquake^45,46^ precludes any surface rupture. Furthermore, very limited changes in local morphology are noticeable between the first aerial photographs dating back from 1964 and post-1988 satellite imagery.

Therefore, the Mw8.3 1934CE earthquake seems most likely to be the earthquake to have ruptured the surface at the Khutti Khola river. Colluvial wedge CW1 deposition can either be associated to the strong shaking in 1934 and/or to the dismantlement of the scarp between 1934 and 1954, a period devoid of large instrumental earthquakes in central Nepal. Rizza et al.^20^ also reported surface ruptures at the Charnath Khola site, 38 km west of the Khutti Khola site. These ruptures were associated by these authors with the 1934 Bihar–Nepal earthquake, based on morphotectonic and paleoseismological observations combined with radiocarbon dating of detrital charcoals. Further west, at the Sir Khola site located at the western extent of the intensity VIII isoseismal, the existence of the surface rupture of the 1934 earthquake is debated^23,24,36^. However, this site is located 63 km west of the Khutti Khola site thus the controversy on the surface rupture there does not impact the results in this paper.

Independently of the paleoseismological studies, Bollinger et al.^24^ and Rizza et al.^20^ have shown that any surface-rupturing event along the MFT in this region would involve an uplift of the hanging wall, which can lead to the abandonment of fluvial terraces. Dating the abandonment of these terraces thus give additional constraints on the timing of the last earthquake rupturing the surface. The terrace T1 associated with F1 has most probably been uplifted during the last earthquake. The detrital charcoals found in the sediments have been sampled 50–100 cm below the surface of T1 and were therefore deposited sometime before the abandonment of the terrace by the river. The highest and youngest detrital charcoal yields a ^14^C age (PIT5-06, Table S1 in the Supplementary Materials) indicating that the sediments at ~ 50 cm were deposited after 1297–1413CE and thus that the terrace was necessarily abandoned later. This result is consistent with an abandonment of a terrace T1 related to the 1934 event.

To the north of F1, T1 was incised by a river, as evidenced by the presence of a perched riverbed becoming the T′1 surface further east. This surface is dated as recent (individually calibrated ages: 1648–1938CE) based on 4 detrital charcoals collected in pit J (PITJ-01,-04,-07,-09, Table S1). This terrace necessarily postdates the seventeenth century. The morphology and age of this channel and the surface T′1 indicate that it could have been deposited either before or slightly after the last event uplifting T1. The formation and abandonment of this channel could be related with the damming of the drainage system by the coseismic fault scarp as well as with the postseismic aggradation and base level changes that followed. A similar scenario associated with post-earthquake river changes and aggradation was previously observed 38 km to the west, at the Charnath site^20^.

These observations suggest major coeval morphotectonic changes along the front in 1934, at least from Sir Khola-Bardibas^24,34^ to Charnath^20^ and Khutti Khola, probably further east^34^ (Fig. 2).

**Figure 7:**
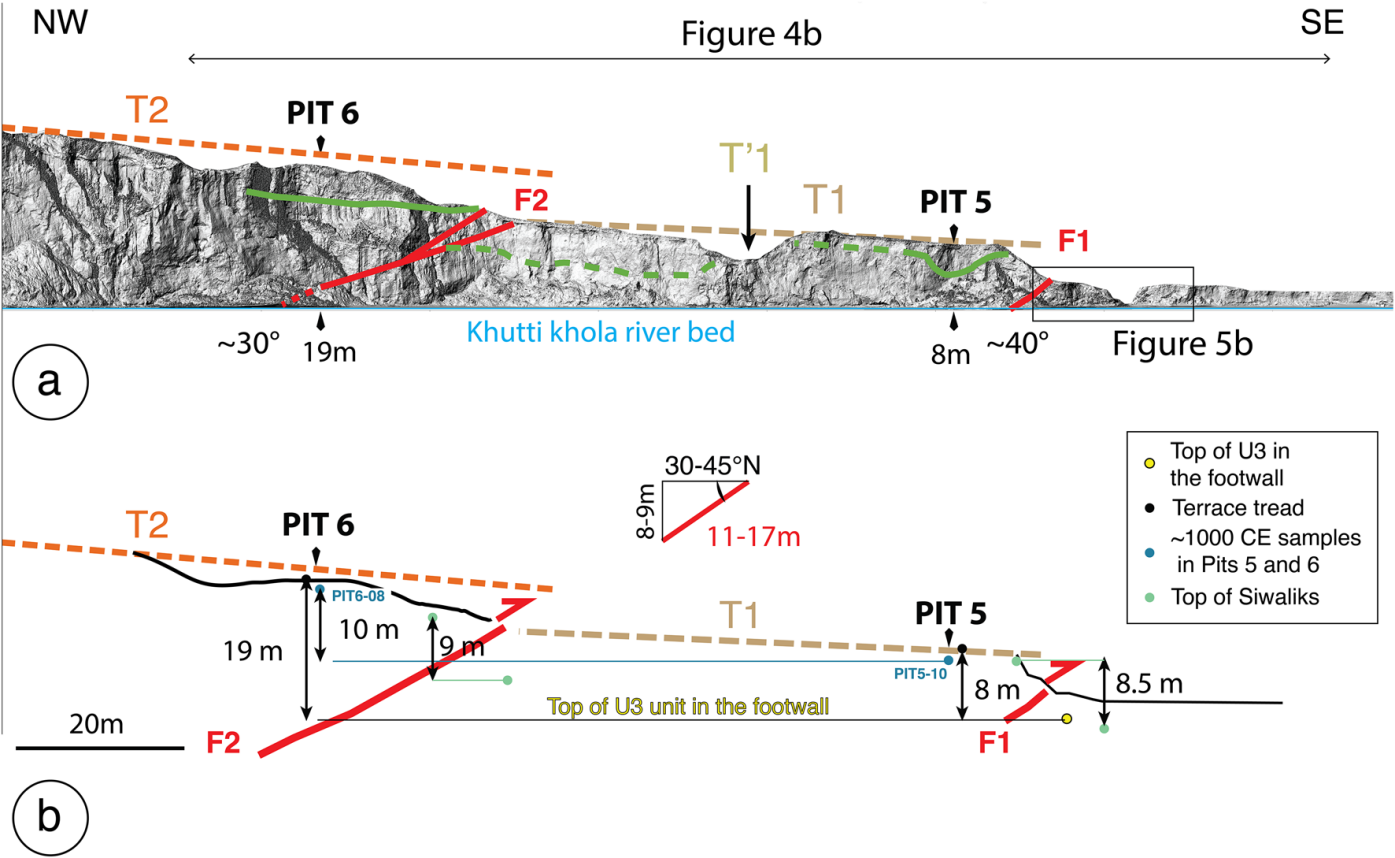
Interpretative section of the Khutti Khola rivercut and coseismic slip. (**a**) Lidar acquired Digital Surface Model of the rivercut. Green lines represent the top of the Siwalik, the black box locates the trench site. (**b**) Schematic section.

As mentioned above (and shown in Fig. 4), a 7 m-high scarp has been mapped along fault F2, and one can find some remnants of a T2 terrace tread in F2 hanging wall. Because the scarp is highly degraded by erosion, we excavated a pit in T2 tread instead of excavating directly the scarp toe. The youngest detrital charcoal in the T2 terrace tread sediments yields a ^14^C age between 903 and 1153 CE (sample Pit6-08, see Fig. 7, Fig. S5). According to earlier observation about F1 and T1, we thus propose that the abandonment of T2 might be related to a surface-rupturing event, uplifting the terrace. Sample Pit6-08 found in Pit6 in T2 terrace indicates that this uplift and the associated surface-rupturing event thus occurred shortly after the tenth to twelfth centuries. According to historical records^43^, this uplift, potentially associated to a medieval earthquake could be related with the 1255 CE earthquake that devastated Katmandu. This earthquake has been associated with (1) a surface rupture on fault F3T and terrace Th4 abandonment at Sir Khola^23,24^ and to (2) PalT4 terrace abandonment at Charnath Khola^20^.

### Offset estimates, distribution of the deformation, and coseismic uplift

In the Himalaya, natural rivercut scarps sites, targeted for paleoseismic studies, are not long-lived features due to the highly contrasting climate of dry winters and rainy summers, which promotes alternation of large erosional and aggradational episodes along river channels. Such climatic conditions tend to rapidly erode the surface expression of past surface ruptures. Only ~ 90 years after the 1934 Bihar–Nepal earthquake, evidence of surface rupture is hard to find, making it difficult to assess coseismic displacement characteristics at the scale of the entire earthquake rupture. Thus, only at a few locations has it been possible to determine a minimum coseismic slip with some confidence, ~ 6 m and between 5.3 and 8.5 m respectively at the Sir and Charnath khola^20,24^.

In this respect, the Khutti Khola site shows valuable evidence of the 1934 rupture. As suggested by the Lidar section in Fig. 7a, the deformation is distributed between two fault zones (F1 and F2). According to their ages, the terraces abandonment seems to be related to two distinct surface-rupturing earthquakes. Even though, we cannot exclude that the deformation associated to the most recent event (MRE) could have been distributed, with F2 accommodating a small part of the displacement, most of this recent displacement seems to have been accommodated by F1. We therefore consider that the two fault zones are associated to two distinct earthquakes. Post-seismic incision and aggradation may conceal part of this tectonic deformation at the rivercut location^20^. Therefore, we use the large-scale morphology of abandoned fluvial terraces within the hanging walls of the fault zones to tentatively estimate coseismic displacements (Fig. 7).

F1 and F1′ both offset the top of the Siwalik by ~ 50 cm, according to the 2012 observations (Fig. 5a). The apparent offset of ~ 50 cm represents a minimum estimate of the slip accommodated by each fault as the top of the Siwalik in the hanging wall is altered and has most probably been eroded. This observation is consistent with what is observed in 2020 where the Siwalik (Us) overthrust the recent deposits U3a by minimum 1 m (Fig. 5b, Fig. S4 in the Supplementary Data material). Even though the 2020 observations cannot constrain properly the slip on F1, which exposed Siwalik rocks trusted over Siwalik rocks, the 2012 wall suggest that the minimum slip accommodated by both faults together is ~ 1 m. Several auxiliary fault splays were found offsetting the Siwalik and the quaternary deposits along the outcrops exposed in 2012 and 2020 between F1′ and F1, as well as below F1 (Fig. 5a,b). This testifies for the internal deformation of the whole Siwalik wedge.

**Figure 8:**
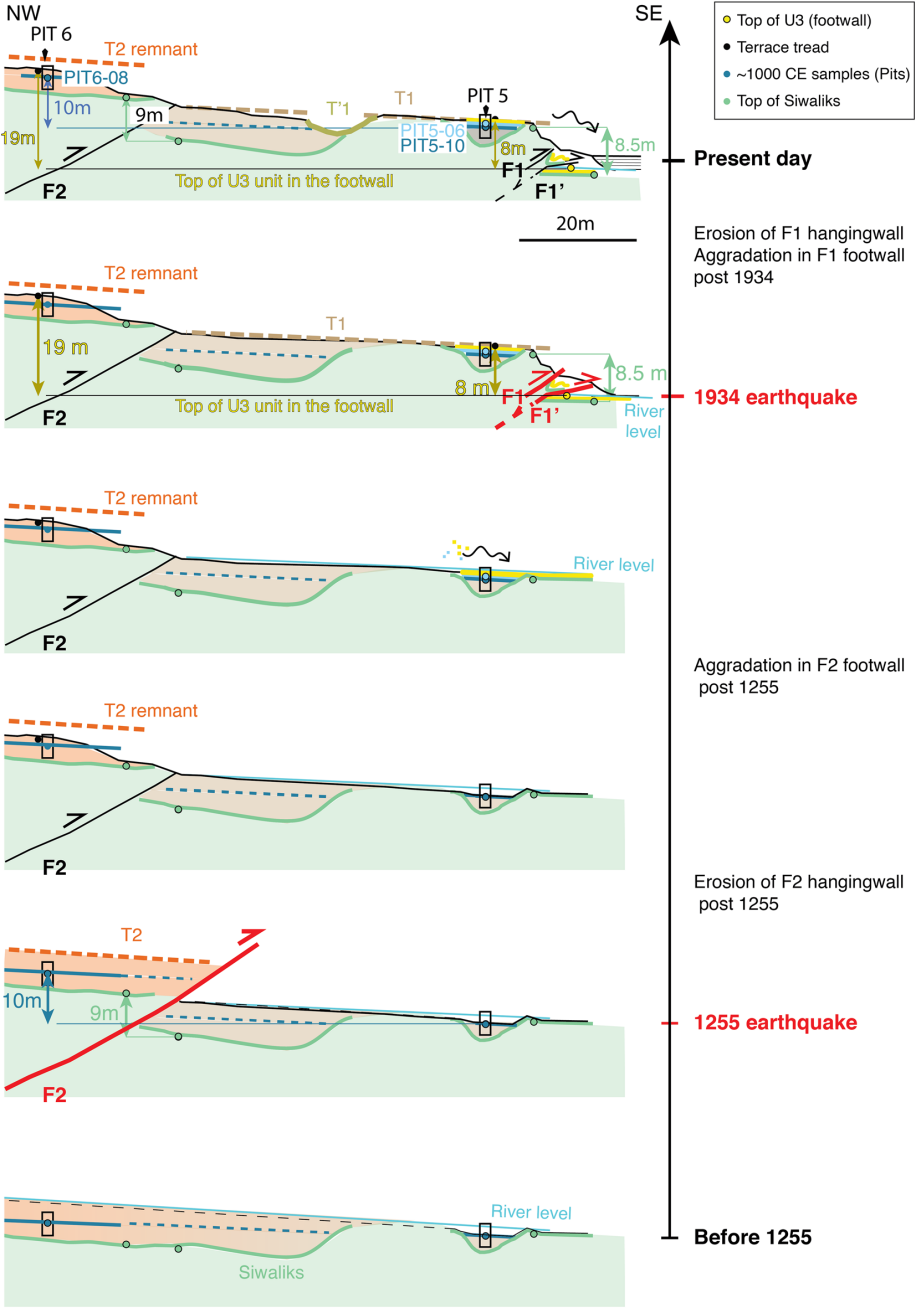
Schematic view of the chronological evolution of the Khutti Khola rivercut.

We subsequently use two separate markers, at the scale of the fault scarps, to estimate the apparent vertical displacement across the fault zones represented Figs. 7 and 8 (in green for the Siwalik and in dark-blue and yellow for the fluvial units).

Across fault F1, the apparent vertical offset between the top of the Siwalik in the southern edge of T1 and the top Siwalik within bottom of the trench bellow F1 is also estimated at ~ 8.5 m (Fig. 7). Another way to estimate the apparent vertical throw of the most recent earthquake is to assume that the ~ 8 m scarp that separates the T1 terrace tread (abandoned after the thirteenth century) and the top of U3 unit in the footwall of the 2020 trench (locating the earthquake horizon), corresponds to the height of the scarp in 1934. In this case, the top of U3 corresponds to the current riverbed level. This apparent vertical throw of ~ 8 m appears to be consistent with the vertical offset of the top of the Siwalik (Figs. 7, 8).

These comparable apparent offsets would require a large coseismic slip in 1934 of ~ 11-17 m provided that the thrust at depth dips between 30 and 45° N^37^, a range of values consistent with the fault dips found in the trench. These observations are also consistent with the dips found at the Sir and Charnath sites^20,23,47^.

Further north, across fault F2, the offset can also be estimated considering the elevation and age of abandonment of the fluvial units. Terrace T2 was abandoned after 903-1153CE, the radiocarbon age of the youngest charcoal sampled ~ 75 cm below the top of T2 in Pit6 (Pit6-08, Fig. S5). This age is similar to the age of the sample Pit5-10 (895–1021CE) collected at a comparable depth (95 cm) in Pit 5 of T1, in the footwall of F2 (blue in Figs. 7, 8). The apparent vertical offset of the fluvial units deposited around the eleventh century in the Khutti Khola river on F2 is therefore approximately 10 m. Furthermore, similarly to the Siwalik offset estimated across F1, a tentative apparent vertical offset of ~ 9 m can be retrieved between the hanging wall and the footwall of F2 by interpolating southward the top of the Siwalik which seems eroded at T2 southern tip (Figs. 7, 8). Although this offset is less constrained, it appears to be consistent with the more robust apparent offset between the fluvial units found in pits 5 and 6.

At the scale of the entire outcrop, the T2 terrace tread stands ~ 19 m above the earthquake horizon of the MRE (U3 unit in 2020 log), if one accounts for the slope of the current river profile. This ~ 19 m-high apparent vertical throw is the result of at least two earthquakes. As previously demonstrated, the most recent one uplifted T1 by ~ 8 m. According to this, the remaining ~ 11 m of apparent vertical throw were therefore cumulated through F1 and F2 between the eleventh century and 1934. This value is comparable with the 2 apparent vertical offsets estimated above in both the fluvial units and the top of the Siwalik of T1 and T2. In the following, it is assumed that the offset measured at the surface is a coseismic offset. This assumption has recently been challenged by studies showing that a significant/measurable amount of thrusting can occur within the decades following large earthquakes on thrusts (e.g.^48^), a feature we cannot resolve here given the poor control on the age of scarp development.

We assume that this apparent vertical throw is related to the penultimate earthquake, and mostly accommodated by F2 as the most recent terrace T1 in its footwall was abandoned later, sometime after the thirteenth century. Considering this and the ~ 30°–45° dip of the MFT estimated from the geological cross section^37^, a coseismic slip of ~ 14–22 m is estimated. This offset can be associated to the penultimate event that occurred necessarily after the tenth century and most probably after the mid-twelfth century, and could possibly be the 1255CE earthquake -considering the ages obtained in the Pit6 excavated in T2. According to our first-order interpretation, the amount of coseismic slip associated with the last two earthquakes seem similar. Although the results suggest a distribution of the deformation progressively propagating southward, one cannot totally discard the possibility that the coseismic slip for both events was distributed across both F1 and F2 fault zones, or that these faults were partly reactivated as out-of-sequence thrusts in association with some faulting located southward, precluding any further interpretation of differences between the two earthquakes. Indeed, several authors have documented the embryonic growth of the fold and thrust belt kilometers south of the Himalayan morphological front (e.g.^49,50^). Locally, at Khutti Khola we clearly in the field evidences for the propagation of the deformation south of F1 along the river course in the emerging Siwalik outcrops. However, we were unable to expose outcrops where the deformation was localized either because they had been swallowed by the river or because they were covered by recent alluvial sediments.

Figure 8 depicts the proposed interpretation for the recent history of the Khutti Khola river catchment aggradation, the seismic events and the southward propagation of the thrust front.

### Slip deficit on the Himalayan megathrust since 1255

The long-term slip deficit on the MFT, and more generally on the up-dip end of the MHT, which is fully locked between earthquakes (e.g.^51^), can be assessed assuming that it corresponds to the interseismic fault slip rate at depth, below the Himalayan range. The interseismic fault slip rate is estimated to ~ 1.5–2.0 cm/year during the decade preceding the Gorkha earthquake, using the Nepal GPS geodetic network^51–54^. Assuming that this rate remained overall constant during the interseismic period, and that the shortening in the hanging wall of the MFT was negligible, the slip deficit between 1255 and 1934 earthquakes can be estimated to ~ 10–15 m. This slip deficit is comparable to the ~ 11–17 m coseismic slip deduced from the fault F1 apparent vertical throw at Khutti Khola. This suggests that most of the interseismic slip deficit accumulated in the region over the last few centuries was probably released seismically during the 1934 earthquake.

Using scaling laws, the moment magnitude of these two earthquakes can be reevaluated based on the new coseismic slip determination^55^. The minimum surface ruptured in 1934, derived from a combination of macroseismic observations and primary surface ruptures, could be estimated around 70 × 80 km (Length (lateral extent) × Width^42^). The minimum slip estimated here is 11 m. If this minimum slip of 11 m is considered to represent the average coseismic slip, it yields a magnitude Mw ~ 8.1, slightly lower than the moment magnitude determined by previous seismological estimates—around Mw8.3–8.4^35,56^. Conversely, the magnitude of Mw8.3 could be reached if one considers a 17 m average slip on a larger surface of 75 × 90 km. The 17 m offset, however, exceeds the amount of slip accumulated between 1255 and 1934. Furthermore, the offset measured at a single site is rarely representative of the average coseismic slip. The 17 m slip measured at the center of the rupture is likely higher than the average slip, given that the estimates on the rupture western termination are significantly lower, as previously described^20,42^. Finally, the rupture lateral extent could have exceeded 75 km from, at least, Sir Khola to Dharan, which are both affected by devastations and extreme landsliding^18,57^. This plausible scenario doubles the surface of the ruptured area. In that case, using our lower bound for coseismic slip at 8–10 m as an average on a surface of 160 × 80 or 90 km (Length × Width), it would be large enough to generate an earthquake with a magnitude Mw8.3, a value consistent with what was proposed based on observations at the Sir Khola as well as with independent seismological observations^35,56^.

## Conclusion

This study shows that the Mw8.3, 1934CE Bihar–Nepal earthquake ruptured the surface at the MFT at the Khutti Khola outlet within the middle of the mesoseismal area. The observations confirm previous findings of primary rupture and terrace uplift observed further west, at Sir and Charnath Khola. At the Khutti Khola site, the local fluvial terraces are interpreted as having been coseismically abandoned in the hanging wall of the most frontal splays of the Main Frontal Thrust. The radiocarbon dating of T2 terrace tread demonstrates that T2 was necessarily abandoned after the tenth century and most probably after the twelfth century. We propose that this terrace abandonment is related with the penultimate surface rupture suggesting that this earthquake happened after the tenth century. According to historical records, this chronological constraint is consistent with the occurrence at this site of the surface rupture of the medieval 1255CE earthquake that devastated Katmandu^34,43^. The apparent vertical offset of the terrace abandoned during the more recent earthquake (MRE, 1934CE) is estimated at ~ 8 m, a value consistent with a co-seismic slip of ~ 11–17 m on the most frontal fault splays. The coseismic slip of the 1934 Bihar–Nepal earthquake is thus comparable to the ~ 10–15 m of slip deficit accumulated between the 1255 and the 1934 surface rupturing earthquakes, indicating that most of the interseismic deformation is released seismically during large surface-rupturing earthquakes.

## Methods

### Mapping of abandoned alluvial terraces

The terraces have been first identified on aerial photographs from the 1960s and then their morphotectonic characteristics have been mapped using helicopter and UAV photographs, Pleiades satellite images and digital surface model (DSM) as well as ground lidar digital elevation model (DEM).

### Digital surface and elevation model generation

We develop new, higher resolution DSMs along the trace of the fault using Pleiades tri-stereoscopic images. These images have a spatial resolution of 0.7 m, and an estimated vertical precision of 1 m.

We then used the Pleiades images to generate a series of Digital Surface Models (DSMs) through an automatic workflow that performs the data co-registration and the DSM generation, as described in Guérin et al.^58^. The data co-registration is based on tie-point detection as a preprocessing step in order to ensure that the images (and hence the DSM) are precisely located. Tie-point detection is particularly sensitive to steep slopes; in order to accommodate this and ensure accurate tie-points, the detection is performed on the images after orthorectification with the most accurate digital terrain model (DTM) available. Following tie-point registration, DSMs are generated for each date, based on so-called ground space image matching with the open-source software MICMAC, which was developed by the French National Geographic Institute (IGN)^59^. This methodology allows an elevation value to be calculated for each point on a grid defined with both planimetric and altimetric steps. The final altitude value is chosen by considering the correlation score and a regularization term^58^. For this study, the DSM was generated with a 2 m planimetric step and 1 m altimetric step.

### Radiocarbon dating of the sedimentary units

The charcoal sampled were prepared and dated at Beta Analytics, Miami, Florida, KECK carbon cycle AMS facility, Irvine, California and SUERC, Glasgow, Scotland.

31 detrital charcoals were dated by AMS (Accelerator Mass Spectrometry) sampled both in the rivercut/trench and in pits excavated in the terrace treads (see Table S1). The samples were individually calibrated using IntCal13 atmospheric calibration curve with Oxcal V4.3^40^. For sample considered as modern (post 1950), we used the Bomb04NH1 calibration curve (Ref.^60^, see Table S1).

The ^14^C calibrated ages predate the deposition age by their in-built age (age of the wood at the time of burning) and the time of transport before deposition^39^. The ages are therefore maxima (older than the actual deposition age). We used stratigraphic information by combining phases (unit with no defined stratigraphic relations) and sequences (unit stratigraphically organized with depth) to place the units in a stratigraphic order in a Bayesian model using the Oxcal software^41^ to refine the chronology of the earthquake horizon) but took into account no inbuilt age nor transport.

## Supplementary Information

Supplementary Information.

## Data Availability

All data generated or analyzed during this study are included in this published article and its Supplementary Information files.
