# Supplementary material for: Surface rupture and landscape response in the middle of the great Mw 8.3 1934 earthquake mesoseismal area: Khutti Khola site
Source: Sci Rep. 2023 Mar 20;13:4566. doi: 10.1038/s41598-023-30697-7 (PMC10027815; doi:10.1038/s41598-023-30697-7)
Supplement: Supplementary file 1 — Supplementary Information. [file 41598_2023_30697_MOESM1_ESM.docx]

**Supplementary materials for**

**Surface rupture and landscape response in the middle of the great Mw 8.3 1934 earthquake mesoseismal area: Khutti Khola site.**

Magali Riesner* (1), now at (2)

Laurent Bollinger (1)

Magali Rizza (2)

Yann Klinger (3)

Çağıl Karakaş (5)

Soma Nath Sapkota (4)

Chanda Basnet (4)

Cyrielle Guérin (1)

and Paul Tapponnier (6)

*correponding author: magali.riesner@gmail.com

(1) CEA, DAM, DIF, F-91297 Arpajon, France,

(2) Aix Marseille Univ, CNRS, IRD, Coll France, CEREGE, Aix‐en‐Provence, France

(3) Université Paris Cité, Institut de Physique du Globe de Paris, CNRS, Paris, France

(4) Department of mines and geology, Nepal

(5) Schlumberger Stavanger Research Center, 4056 Tananger, Risabergvegen 3, Norway

(6) Institute of Crustal Dynamics, China Earthquake Administration, Beijing, China

Table S1: AMS Radiocarbon (14C) Dates for detrital charcoals sampled in the terraces and scarp.

| **Sample number** | **Location** | **Measured radiocarbon age (Years BP) ^b^** |  | **Calibrated Ages (calendric,2S)^c^** |
| --- | --- | --- | --- | --- |
| KKR20-10 | Tributary channel 2020 | Modern Fraction 1,1367 ± 0,0030 | Intcal13+NHZ3 | 1958-1994 CE |
| KKR20-09 | Tributary channel 2020 | Modern Fraction 1,1229 ±0,0028 | Intcal13+NHZ3 | 1957 CE |
| KKR20-18 | Rivercut Wall 2020 | 2120+/-260 | Intcal13 | 802 BCE-401 CE |
| KKR20-02 | Rivercut Wall 2020 | 1080+/-45 | Intcal13 | 779*-1030 CE |
| KKR20-15 | Rivercut Wall 2020 | 7690+/-600 | Intcal13 | 8226-5540 BCE |
| KKR20-12 | Tributary channel 2020 | Modern Fraction 1,0539 ± 0,0027 | Intcal13+NHZ3 | 1957 CE |
| KKR20-08 | Rivercut Wall 2020 | 170+/-25 | Intcal13 | 1662-1917 CE |
| KKR20-19 | Rivercut Wall 2020 | 280+/-100 | Intcal13 | 1439-1914 CE |
| KKR20-11 | Tributary channel 2020 | 101.38 +/- 0.38 pMC | Intcal13+NHZ3 | 1954-1956 CE |
| KKR20-05 | Rivercut Wall 2020 | 140 ± 30 BP | Intcal13 | 1669-1944 CE |
|  |  |  |  |  |
| KKR12-01 | Rivercut Wall 2012 | 66 ± 34 | Intcal13 | 1690-1926 CE |
| KKR12-02 | Rivercut Wall 2012 | 194+/-23 | Intcal13 | 1657-1954 CE |
| KKR12-13 | Rivercut Wall 2012 | Modern Fraction 1,1229+/-0,0031 | Intcal13+NHZ3 | 1666-1951 CE |
| KKR12-18 | Rivercut Wall 2012 | 152+/-23 | Intcal13 | 1666-1951 CE |
| KKR12-31 | Rivercut Wall 2012 | Modern Fraction 1,0049+/-0,0032 | Intcal13+NHZ3 | 1954-1956 CE |
| KKR12-38 | Rivercut Wall 2012 | 120+/-26 | Intcal13 | 1680-1764 CE |
| KKR12-34 | Rivercut Wall 2012 | 123 ± 34 | Intcal13 | 1679-1941 CE |
| KKR12-28 | Rivercut Wall 2012 | Modern Fraction 1.0039 ± 0.0043 | Intcal13+NHZ3 | 1952-1954 CE |
| KKR12-42 | Rivercut Wall 2012 | 200 ± 34 | Intcal13 | 1644-1955 CE |
| KKR12-50 | Rivercut Wall 2012 | 163 ± 34 | Intcal13 | 1662-1953 CE |
|  |  |  |  |  |
| KKR12-08 | Rivercut channel | 1245+/-26 | Intcal13 | 682-870 CE |
| PIT5-01 | Pit 5 | 1196 ± 34 | Intcal13 | 695-945 CE |
| PIT5-06 | Pit 5 | 593 ± 34 | Intcal13 | 1297-1413 CE |
| PIT5-10 | Pit 5 | 1072 ± 34 | Intcal13 | 895-1021 CE |
| PIT6-03 | Pit 6 | 1081 ± 34 | Intcal13 | 893-1018 CE |
| PIT6-05 | Pit 6 | 1697 ± 34 | Intcal13 | 255-416 CE |
| PIT6-08 | Pit 6 | 1011 ± 34 | Intcal13 | 903-1153 CE |
| PITJ-1 | Pit J | 85 ± 30 | Intcal13 | 1688-1927 CE |
| PITJ-4 | PITJ | 90 ± 30 | Intcal13 | 1684-1929 CE |
| PITJ-7 | PITJ | 110 ± 30 | Intcal13 | 1681-1938 CE |
| PITJ-9 | Pit J | 125 ± 30 | Intcal13 | 1677-1940 CE |

aSamples have been dated by accelerator mass spectrometry (AMS) measured at Irvine /SUERC Glasgow and Beta Analytics AMS facilities. Each number corresponds to the laboratory code for each sample.

bConventional Radiocarbon years B.P. relative to 1950 A.D. (with 1 s confidence level including counting statistics as well as reference standard, blank and random machine errors).

cCalendric dates were calibrated using the atmospheric calibration curve IntCal13 (for the Northern Hemisphere [Reimer et al., 2013].


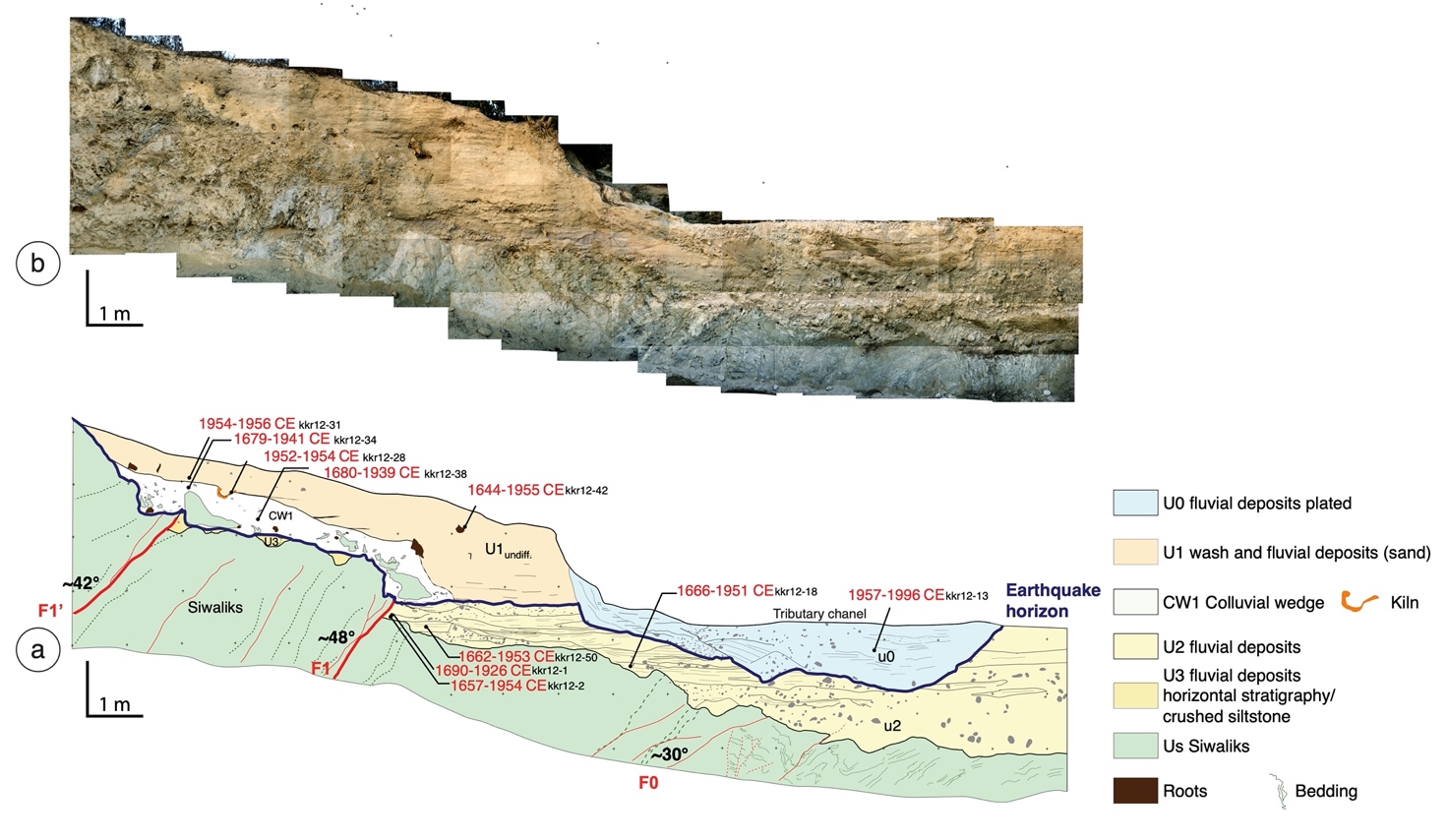


Figure S1: (a) Orthophotomosaic of the 2012 rivercut/trench and (b) log of the trench


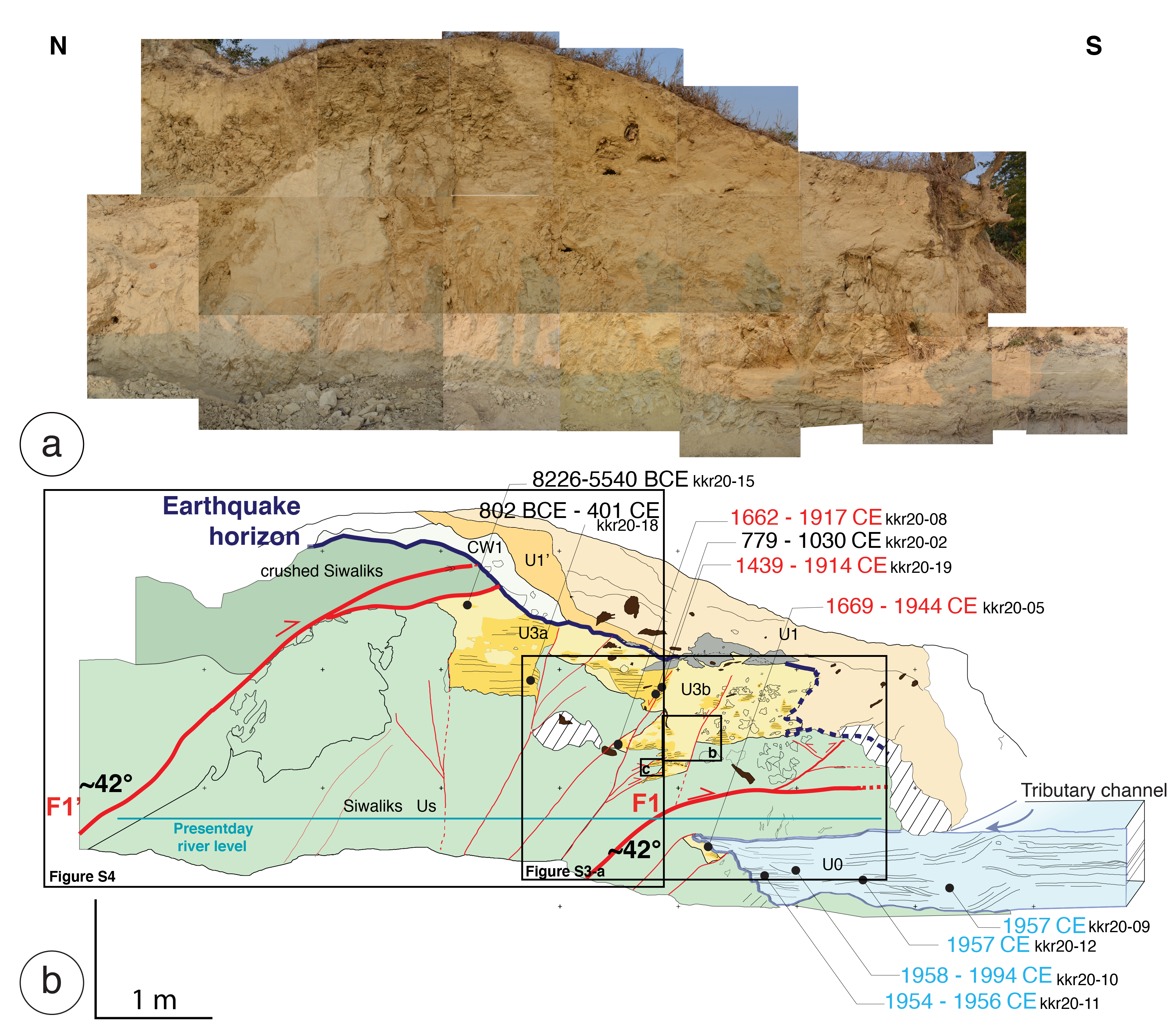


Figure S2: (a) Orthophotomosaic of the 2020 rivercut/trench and (b) log of the trench. Black boxes locate Figures S3 and S4.


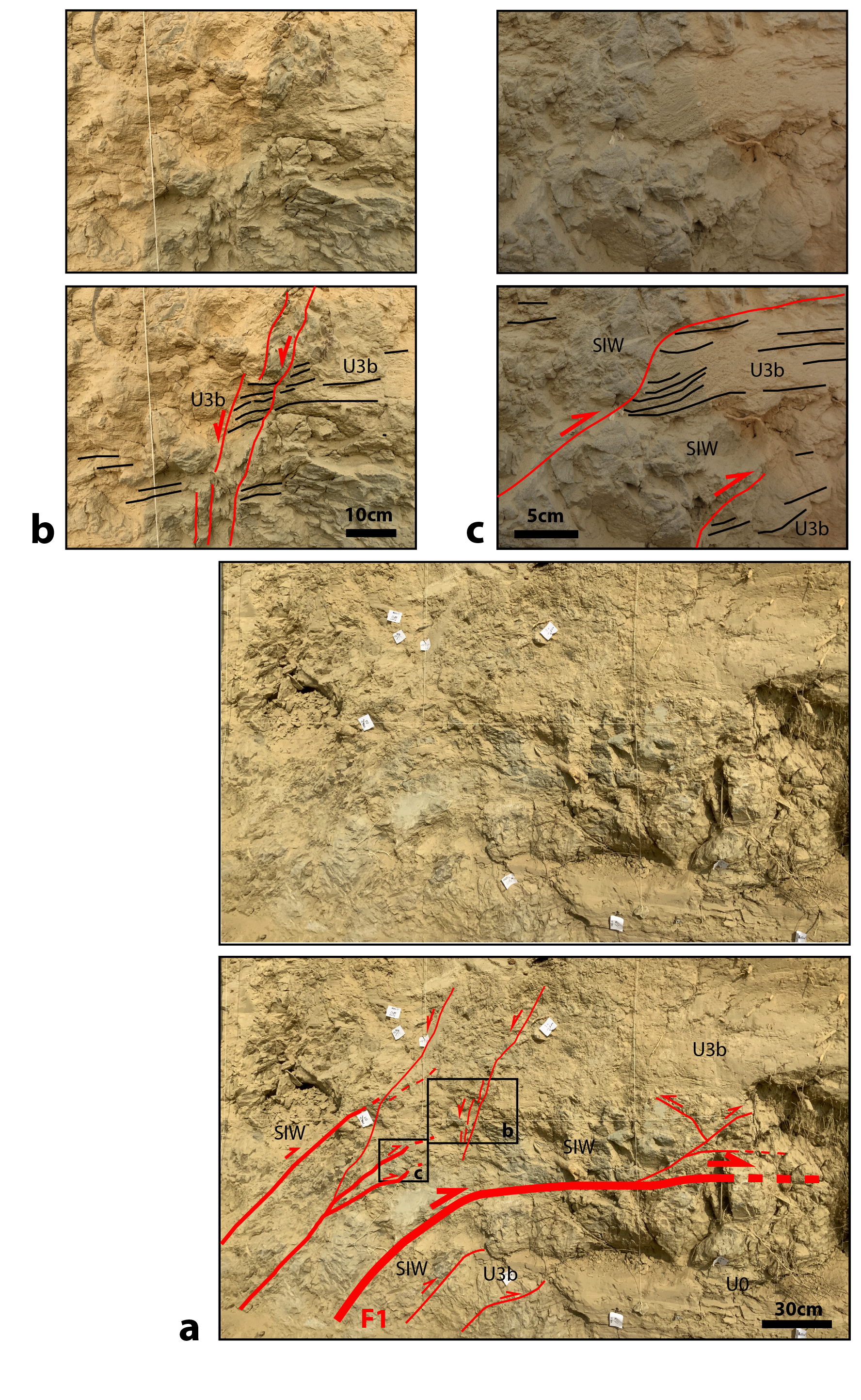


Figure S3: Detailed views on the deformation. Raw images (top) and interpretated structures (bottom). Photos are located on the log Figure S2.


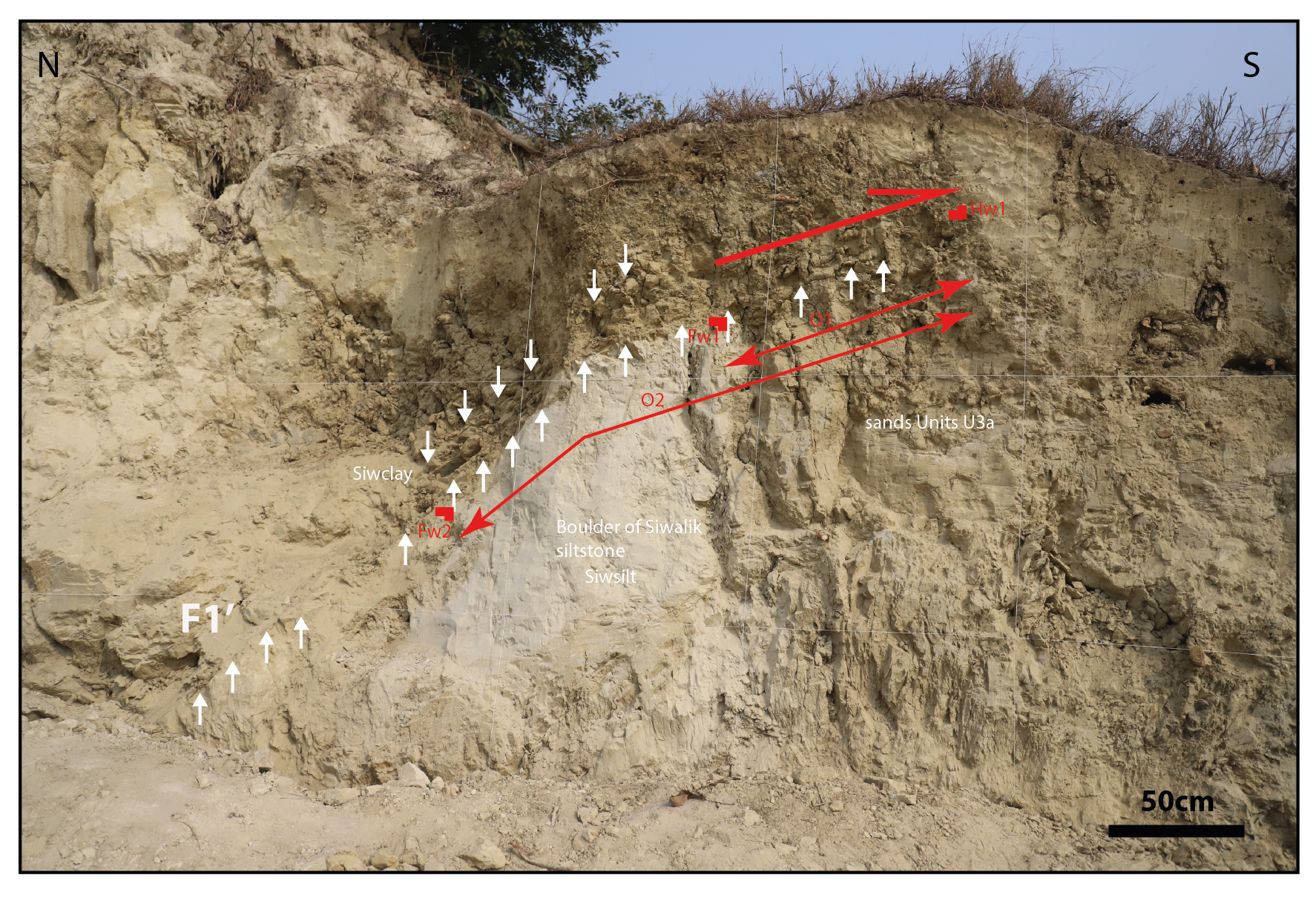


Figure S4: Photo taken in January 2019 of fault F1’, affecting the northern part of the Khutti Khola scarp along the natural river cut.  The main shear zone is delimited / underlined by the white arrows. Hw and Fw locate markers respectively in the footwall and Hangingwall of the thrust: Hw1 correponds to the trailing edge of the clayish Siwalik unit transported over the main boulder of Siwalik siltstone and unconsolidated sandy units U3a. Fw1 materialize the edge of the siltstone boulder. O1, materialized by the double headed red arrow is the minimum offset along fault F1’ (around 1 meter) assuming that the claystones of the trailing edge of the thrust fault was at Fw1 before the earthquake. Another scenario, in which we assume that the claystone in the hangingwall of F1’ are correlated with the claystones found at Fw2, the top level of claystones in the footwall would require a larger offset O2 on F1’ (2.4 m), but necessitate that the boulder of Siwalik siltstone was an element of the topography before the earthquake. The photo is located on Figure S2.


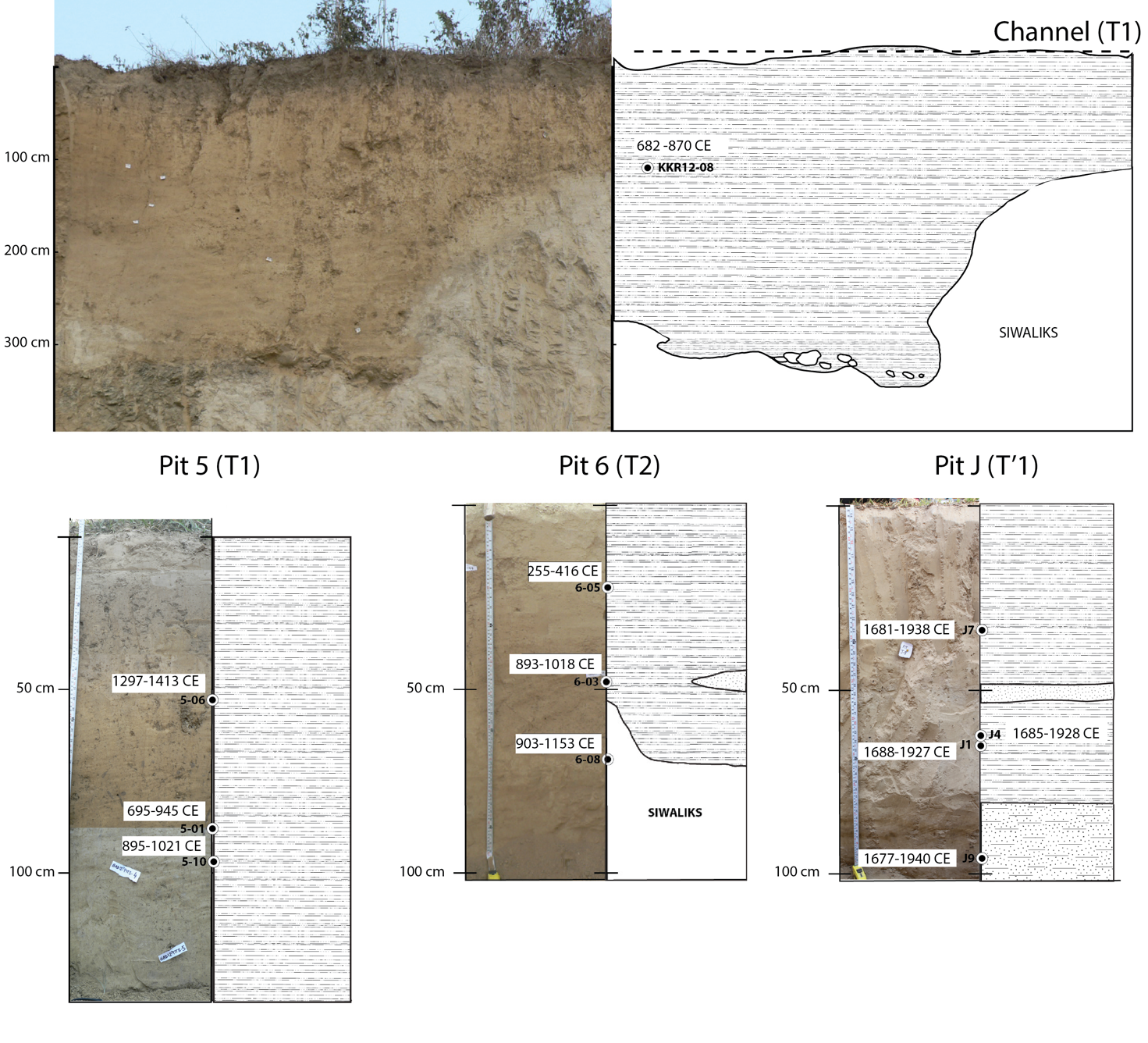


Figure S5: Stratigraphic log of the Pits located on Fig. 3a and the channel on the rivercut on T1 located on Fig. 3b.
